# Rapid Screening of New Psychoactive Substances Using pDART-QqQ-MS

**DOI:** 10.1021/jasms.4c00124

**Published:** 2024-04-23

**Authors:** Wei-Hsin Hsu, Kai-Wen Cheng, Tzu-Hsuan Feng, Ju-Yu Chen, Guan-Yuan Chen, Lian-Yu Chen, Te−I Weng, Cheng-Chih Hsu

**Affiliations:** † Department of Chemistry, National Taiwan University, Taipei 10617, Taiwan; ‡ Forensic and Clinical Toxicology Center National Taiwan University College of Medicine and National Taiwan University Hospital, Taipei 10051, Taiwan; § Department and Graduate Institute of Forensic Medicine, College of Medicine, National Taiwan University, Taipei 10051, Taiwan; ∥ Institute of Epidemiology and Preventive Medicine, National Taiwan University, Taipei 10051, Taiwan; ⊥ Kunming Prevention and Control Center, Taipei City Hospital, Taipei 108203, Taiwan; ∞ Leeuwenhoek Laboratories Co. Ltd., No. 71, Fanglan Rd, Taipei, 106038, Taiwan

## Abstract

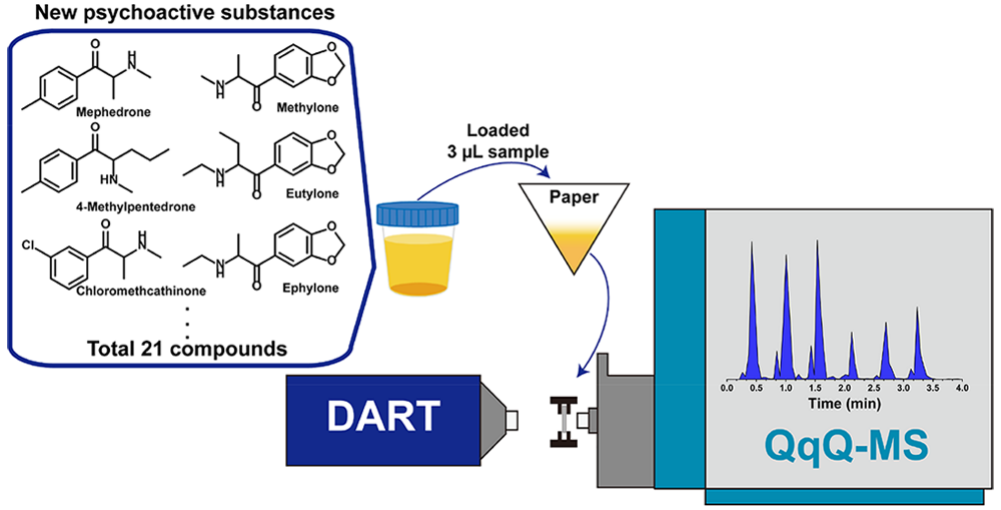

Drug abuse is a severe social problem worldwide. Particularly, the issue of new psychoactive substances (NPSs) have increasingly emerged. NPSs are structural or functional analogs of traditional illicit drugs, such as cocaine, cannabis, and amphetamine; these molecules provide the same or more severe neurological effects. Usually, immunoassays are utilized in the preliminary screening method. However, NPSs have poor detectability in commercially available immunoassay kits. Meanwhile, various chromatography combined with the mass spectrometry platform have been developed to quantify NPSs. Still, a significant amount of time and resources are required during these procedures. Therefore, we established a rapid analytical platform for NPSs employing paper-loaded direct analysis in real time triple quadrupole mass spectrometry (pDART-QqQ-MS). We implemented this platform for the semiquantitative analysis of forensic drug tests in urine. This platform significantly shrinks the analytical time of a single sample within 30 s and requires a low volume of the specimen. The platform can detect 21 NPSs in urine mixtures at a lower limit of qualification of concentration ranging from 20 to 75 nanograms per milliliter (ng mL^–1^) and is lower than the cutoff value of currently available immune-based devices for detecting multiple drugs (1000 ng mL^–1^). Urine samples from drug addicts have been collected to verify the platform’s effectiveness. By combining efficiency and accuracy, our platform offers a promising solution for addressing the challenges posed by NPSs in drug abuse detection.

## Introduction

The abuse of new psychoactive substances (NPSs) is a major issue in modern society. NPSs, also known as “designer drugs” or “legal highs,” refer to emerging compounds designed and manufactured with the intent of mimicking existing drugs and circumventing legislative measures enforced by law enforcement.^1^ Over the past decade, regulatory challenges and health risk concerns related to NPSs have dramatically risen due to their widespread proliferation. Traditionally, the quantitative confirmation of NPSs in biological matrices has been carried out using hyphenated mass spectrometry with chromatography techniques in forensic laboratories.^2−4^ These approaches not only provide the sensitivity and accuracy necessary for screening analysis of many NPSs, but they have also led to the publication of methodologies for measuring specific emerging drug classes and a broad spectrum of drugs from different classes.

Normally, law enforcement agencies rely on immunoassays as the preliminary screening method. Immunological tests work based on specific antibodies that bind to targeted substances.^5^ Unfortunately, most commercially available immunological devices are limited to a set number of well-studied and long-existing drugs. On the other hand, emerging legal highs, which drug designers skillfully design, need better detectability with the existing immunoassays on the market. The development of immune-based screening kits for NPSs cannot keep up with the rapid emergence of novel NPSs due to the challenges in advancing specific binding antibodies. Therefore, efficient and high-selectivity approaches to screening are needed for forensic analyses.

Mass spectrometer analytical platforms have been considered to hold great potential for forensic applications.^6,7^ Specifically, ambient ionization mass spectrometry (AIMS) involving the direct sampling and ionization of analytes in ambient air has been thrivingly introduced and developed in several scientific fields.^8^ A variety of methods using different AIMS techniques have been developed for the rapid detection or quantification of NPS.^9−11^ Of these, direct analysis in real time (DART) is a plasma-based ion generation technique operating in a native environment.^12^ Its efficient ionization of nonpolar small molecules makes it suitable for various forensic applications.^13^ While DART-MS has been extensively studied for analyzing seized drugs, its implementation on biological samples is limited due to the requirement of pretreatment, which complicates the analysis process.^14−16^ To overcome this limitation, researchers have investigated the coupling of extraction techniques with DART for samples composed of complex matrices, such as urine, blood, and oral fluid.^17,18^ Although solid-phase microextraction (SPME)-DART-MS has shown promising results for toxicological analyses, it still required several hours (1.5–4 h) to complete the procedures.

As a solution, we developed the paper-loaded DART strategy, (pDART), for rapid screening. The pDART approach utilizes commercial DART-MS coupled with paper-loaded dried samples for rapid mass spectrometric analysis. Previous studies have well-characterized its application in quantifying endogenous serum metabolites and biological short-chain fatty acids.^19,20^ Thus, we proposed a preparation-free, high-throughput semiquantitative pDART coupled with triple quadrupole MS for measuring urine illicit drugs within 30 s per sample. This study described the development and validation of the platform with the purpose of its application in a forensic setting. Following international guidelines,^21^ a validation protocol was applied to evaluate the applicability of the pDART drug screening platform, examining its effectiveness on real samples.

## Materials and Methods

### Materials and Reagents

Ketamine, methoxetamine, norketamine, deschloroketamine, mephedrone, 4-MPD, MEAP, CMC, methylone, ephylone, eutylone, 3,4-MDPHP, dibutylone, MDPV, α-PVP, 4-chloro-α-PVP, amphetamine, methamphetamine, 6-acetylmorphine, MDA, MDMA, DMA, PMEA, PMA, and PMMA were selected as target analysts due to the popularity of these NPSs in the authors’ region. All substances, both the NPSs and opiate metabolite, and isotope-labeled ketamine-D_4_ and methamphetamine-D_8_ (used as the internal standards, ISs) were obtained from Cerilliant (Round Rock, TX, USA) at concentrations of either 100 μg mL^–1^ or 1 mg mL^–1^ in methanol. TOYO no. 50 chromatography paper (Advantec, TOYO, Tokyo) was used as the sample loading material. Ultrapure water (18.2 MΩ cm) was prepared using an Elga system (Elga LabWater, High Wycombe, UK). LC–MS grade methanol was purchased from Duksan Pure Chemicals (Ansan, Korea).

### Biological Sample Collection

Blank urine samples were obtained from seven healthy volunteers, including three males and four females, as the matrix blank. Additionally, 40 urine samples were collected from intoxicated patients who visited the psychiatric emergency department at the Songde Branch of Taipei United Hospital. All the above samples were collected under the institutional review board protocol and approved by the ethics committee of the Ethics Committee of Taipei City Hospital (TCHIRB-10903020). All specimens were anonymized to protect the subjects’ privacy by removing any identifiable information. Neat urine specimens were collected in sample collectors and stored at −20 °C until screening. No further sample pretreatment was conducted on any specimens except for vortexing.

### Sample Preparation

All test solutions, including urine specimens and spiked standard solutions with the desired levels of analyzed compounds, were mixed with deuterated internal standards. The final concentration of the deuterated internal standards was kept constant in all the samples and matrix blanks at 90.91 ng mL^–1^. For the calibration curve, a 100 μL mixture of the standard solution was collected, and 10 μL was pipetted into the matrix blank and then thoroughly mixed. The chromatography paper was cut into equilateral triangular pieces (each side measuring 1.0 cm) using a paper punch and then fixed on the transmission module. The working solution was carefully loaded (0.75 μL) onto the apex of the paper triangle and left undisturbed until it dried. This process was repeated four times, resulting in a final loading volume of 3 μL. Once the paper-loaded samples were dried, the module was installed onto the axial translational motor to analyze. During the real sample analysis, only the deuterated internal standards were added and vortexed. All subsequent steps were the same as those of the calibration curve.

### pDART-QqQ-MS

A DART SVP ion source (IonSense, Saugus, MA) interfaced with the mass spectrometer via a VAPUR interface (IonSense, Saugus, MA) was utilized for all the pDART-MS experiments in this study. Ultrahigh purity nitrogen (99.999%) served as the standby gas, while ultrahigh purity helium (99.999%) was used as the running gas. Both nitrogen and helium output pressures were set at 0.5 MPa, and the grid voltage was set at 250 V in positive mode. The gas heater temperature of the helium reagent gas was adjusted depending on the specific experiment. An automatic transmission module (IonSense, Saugus, MA) was used as a paper holder, carrying ten paper triangles sliced by a commercial paper cutter in one set of experiments. The scanning of ten samples was completed in about 6 min, with a measurement rate of 0.4 mm/sec.

All experiments were performed using the SCIEX QTRAP 5500 System (AB SCIEX 5000 QTRAP, Toronto, Ontario, Canada). The mass spectral analysis and data were collected and processed with the software package of Analyst 1.6.3 (AB SCIEX). The quantitative validation of drugs of abuse and real sample analysis were conducted in the multiple reaction monitoring (MRM) mode with positive ionization. All analyte parameters were collected under flow injection analysis with an ESI resource. The MS inlet capillary temperature and voltage were maintained at 300 °C and 35 V, respectively. Before application on paper-loaded DART analysis, isobaric interferences on chromatography paper were assessed to evaluate the selectivity of the transitions. Two to four transitions were selected for each compound, and the optimized declustering potential (DP), entrance potential (EP), collision energies (CE), and collision cell exit potential (CXP) are summarized in Table S1.

### Data Processing

The area under the curve (AUC) of the analyte fragment ion over the entire analysis time was determined by manual peak integration using Sciex OS (AB SCIEX). In addition, the Kruskal–Wallis nonparametric test with Dunn’s post-test and one-way ANOVA followed by Dunnett’s multiple comparisons test were performed using GraphPad Prism version 9.20 for Windows (GraphPad Software, San Diego, California, USA, www.graphpad.com).

### Validation of Forensic Toxicological Methods

The validation studies followed the standard practices and recommendations from the Scientific Working Group for Forensic Toxicology (SWGTOX) for Method Validation in Forensic Toxicology. Blank matrices obtained from pooled drug-free volunteers’ urine collection were used in all validation parameters, including linearity, accuracy, precision, etc. Multiple-point calibration standards for the 21 analytes were prepared over a concentration range from 1 to 1000 ngmL^–1^ (1, 5, 10, 20, 50, 75, 100, 200, 400, 500, 750, 1000 ng mL^–1^). Details of the calibration curve and validation were provided in the Supporting Information.

### Method Comparison

All 40 anonymous urine specimens were run in triplicate alongside the same test set. The results of this pDART drug screening platform were compared to those obtained from an LC–MS/MS confirmatory assay, as described in a previous study.^22^

## Results and Discussion

### pDART-QqQ-MS Development

The platform was optimized in terms of ionization helium temperature, DP, CE, and scan speed. The DP and CE values were optimized using the ESI ionization source in positive mode and confirmed by the DART resource for fragmentation. The gas temperature was set as low as possible while still producing a stable signal, and lower voltages were preferred to reduce interference from unexpected thermal degradation. The ionization temperature was adjusted empirically, and it was found that 300 and 400 °C acquired adequate intensity for different NPS, probably due to the degradation of the molecules.^23^

Notably, the preliminary experiments revealed a strong background signal in the matrix blank, which was a urine mixture pooled from multiple drug-free human samples to simulate real testing scenarios (Figure S1). To mitigate the background noise, the characteristics of papers as loading materials were investigated.^24^ Biological samples were loaded onto chemigraphic paper using multiple drying and sampling processes to enhance the overall loading volume, capturing and preconcentrating the analytes while absorbing the matrix into the paper. To improve the platform’s sensitivity, we optimized the sample loading volume by testing different volumes ranging from 0.75 to 6.75 μL to enhance the signal-to-noise ratio.

**Figure 1:**
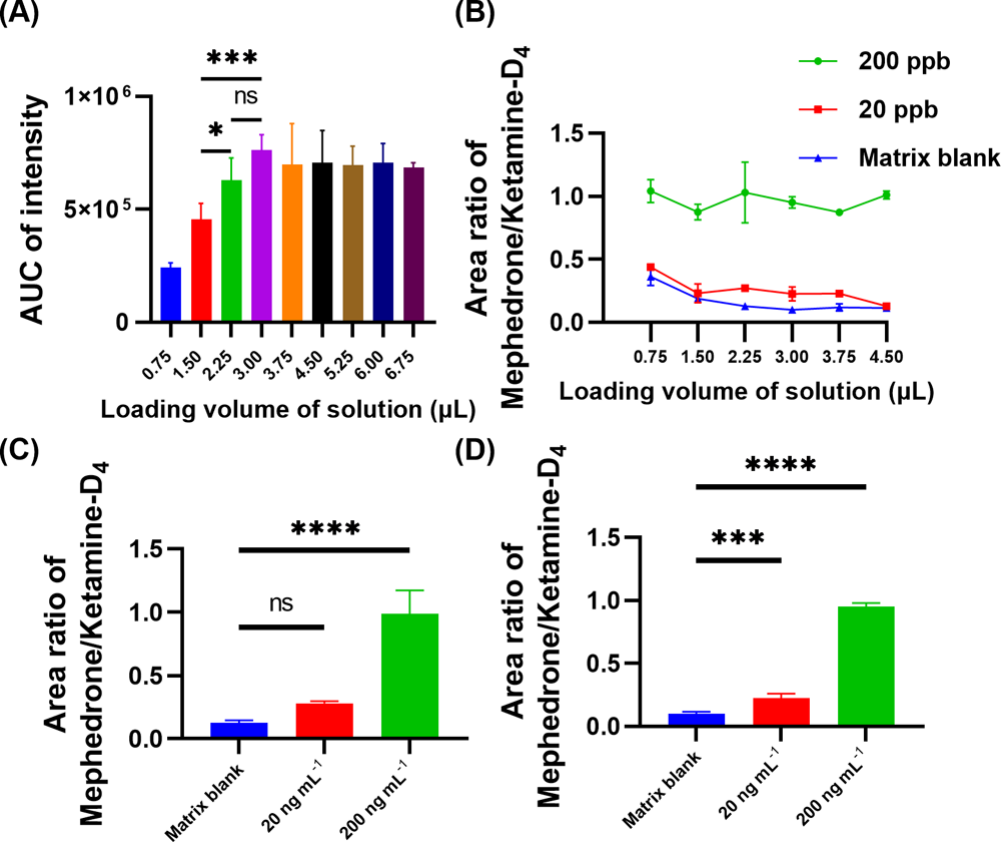
Optimization of sample volume. (A) We evaluated the AUC of ketamine-D_4_ intensity in the matrix blank solution at different loading volumes, ranging from 0.75 to 6.75 μL. (B) We assessed the area ratio of mephedrone/ketamine-D_4_ in the matrix blank solution at loading volumes of 20 and 200 ng mL^–1^, ranging from 0.75 to 4.5 μL, to determine the signal-to-noise ratio. (C) Loading volume was set at 2.25 μL for the matrix blank, 20 ng mL^–1^, and 200 ng mL^–1^ mephedrone. (D) Similarly, the loading volume was 3.0 μL (*n* = 3, *q < 0.05, ** *q* < 0.01, *** *q* < 0.001, and **** *q* < 0.0001 from ANOVA-Tukey analysis.).

In Figure 1A, we plotted the overall sampling volume against the AUC of the ketamine-D_4_ transition. We found that the intensity was highest and most stable when the total solution volume was 3.0 μL. After drying, the final amount of ketamine-D_4_ that loaded onto the paper was 272.73 ng. Paper-based analytical devices have certain properties that affect their ability to retain particles, their pore size, basis weight, and thickness. These properties ultimately determine the capacity and spreadability of liquid samples on the paper.^25^ Hence, we think when the sample loaded 3 μL, the pore size of the paper was saturated, resulting in the subsequent tendency to stabilize. Afterward, we introduced mephedrone (*m*/*z* 178 to 160) at different concentrations (20 and 200 ng mL^–1^) to examine the signal intensity by testing different volumes ranging from 0.75 to 4.5 μL (Figure 1B). We observed a significant difference (*p*-value < 0.01) in signal intensity between the matrix blank and the loaded volume of 3 μL (Figure 1D), while loading 2.25 μL showed no significant difference (Figure 1C) in the matrix blank and 20 ng mL^–1^.

**Figure 2:**
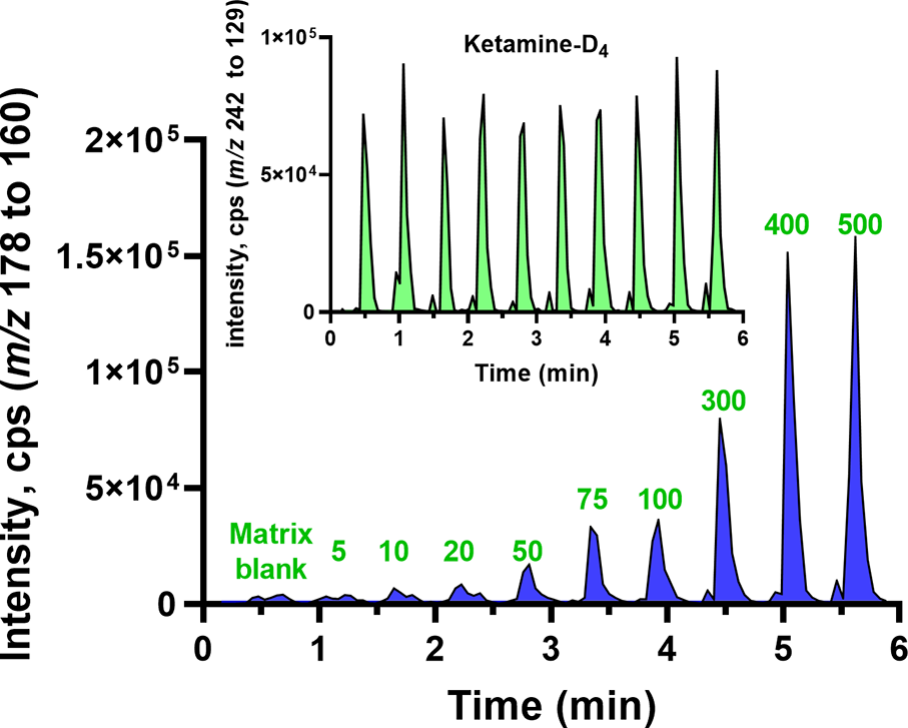
Extracted ion chromatogram (XIC) of mephedrone (*m*/*z* 178 to 160) was analyzed from the matrix blank and samples with low to high concentrations (5 to 500 ng mL^–1^). The XIC includes the internal standard ketamine-D_4_ (*m*/*z* 242 to 129).

By increasing the overall sampling volume on the paper, we significantly improved the sensitivity of the methodology. Consequently, the peak areas of mephedrone corresponding to different concentrations, ranging from low to high, were substantially amplified. Furthermore, this adjustment effectively stabilized the intensity of the internal standard (ketamine-D_4_), as demonstrated in Figure 2.

### Analytical Performance and Validation

Before applying an analytical platform in forensic casework, it is essential to ensure their reliability, robustness, and accuracy through analytical validation. This validation data helps test the strength and suitability of the strategy. To assess the real-time screening performance in real-world scenarios, validation was conducted using the spiked-in pooled urine blank. The quantitative approach involved adding the internal standard mixture directly to the sample collector before sampling.

Selectivity is a crucial aspect of the analytical platform, and thorough validation of this parameter is necessary to prevent false-positive findings, especially when no separation methods are applied prior to MS analysis. To assess the platform’s specificity toward endogenous compounds, urine samples and double blanks from 4 drug-free females and 3 drug-free males were analyzed. No unexpected interference was observed in all analytes, as MRM transitions were used for each analyte, demonstrating a good selectivity of the platform, such as MRM of synthetic cathinones (Figure S2). Notably, some interference in the double blank (not containing internal standard nor analyte) occurred with the internal standard ketamine-D_4_ at a gas temperature of 400 °C; therefore, methamphetamine-D_8_ replaced ketamine-D_4_ as the internal standard in the analytes tested at 400 and 300 °C, respectively.

Linearity is constructed to evaluate the suitability of the quantitative approach. The testing range for linearity spanned 3 orders of magnitude. Good linear fits were obtained for all the analytes under this scenario in the *R*^2^ values ranging from 0.8343 (amphetamine) to 0.9963 (PMMA) (Table S2). Sensitivity was assessed by limits of detection (LOD) and limits of quantitation (LOQ). Below 40 ng mL^–1^, the calculated LODs of most targeted drugs achieved the sensitivity for a toxicological application. The exemptions were amphetamine and 6-acetylmorphine reported at the level of 113.33 ng mL^–1^ and 66.39 ng mL^–1^, respectively. In general, urine as a matrix can result in high background and potential interferences,^26^ which may be a reason for poor linearity or calculated LODs of amphetamine and 6-acetylmorphine. The concentration ranges for the platform’s LLOQ were from 20 to 75 ng mL^–1^, and the LLOQ is determined as the lowest concentration of standard at which the bias is within ±15%. Accuracy, precision, and other validation features were tested at different concentrations, including LLOQ, 400, and 750 ng mL^–1^ for each analyte. The detailed results of the validation are reported in Table S2. No carryover effect was observed in the experiment after treating the upper concentration of 1000 ng mL^–1^ used to make the calibration curves. This result is consistent with the noncontact fashion of DART ionization, which exhibits minimal memory effects.

With regard to accuracy, biases were within ±15% at each level, except for amphetamine, which showed a bias of 54.38% at 75 ng mL^–1^ but was acceptable at the medium concentration (400 ng mL^–1^). The methodology demonstrated good precision, with %CV values below 20% for all species ionized at 300 °C at the LLOQ level. However, the deviation of two species, 6-acetylmorphine and MDA, analyzed at 400 °C, exceeded the criterion level. The results and the high calculated LOD of 6-acetylmorphine might be caused by potential chemical interference at a higher ionization temperature.

The test solutions were considered stable based on the interday %CV value falling within ±30% and bias value limited to ±30% at each concentration. With regard to the matrix effect, due to a lack of separation or sample purification, intense results were expected. Most compounds showed a negative matrix effect, indicating ion suppression when samples were prepared in urine compared to 50% methanol. However, four analytes, namely norketamine, amphetamine, 6-acetylmorphine, and PMA, presented positive matrix effects of approximately 140% at the LLOQ level. In summary, this platform exhibited generally good performance during validation.

### Evaluation on Real Samples by pDART-QqQ-MS

We analyzed 40 urine samples collected from drug-abused subjects using a validated method. Each sample’s prediction was assessed in triplicate, and the result were presented in Table S3. Overall, the signal stability was found to be less than 20%, except for some compounds with relatively low concentrations, which exhibited larger CV%.

In the pDART screening results, the compound was identified as positive if it was present and negative if it was absent. When the compound was analyzed using the LC-QqQ-MS method, it was identified as true negative (TN) by both methods when it was absent and true positive (TP) by both methods when it was present. However, there were instances where the pDART screening results identified the compound as present, but the LC-QqQ-MS method did not detect it, leading to a false positive (FP) result. Conversely, when the compound was absent during the pDART screening test but was detected by the LC-QqQ-MS method, it resulted in a false negative (FN) outcome. The qualitative results of the pDART platform and the comparison with LC-QqQ-MS were presented in Table 1.

**Table 1:** Qualitative Comparison of the pDART Screen with LC-QqQ-MS was Conducted in 40 Urine Specimens^a^

	Predict (pDART-QqQ-MS)		LC-QqQ-MS				Performance		
Compound	Positive	Negative	TP	FP	TN	FN	PPA	NPA	OPA
Ketamine	16	24	14	2	22	2	87.50%	91.67%	90.00%
Methoxetamine	0	40	0	0	40	0	NA	100.00%	100.00%
Norketamine	21	19	17	4	18	1	94.44%	81.82%	87.50%
Deschloroketamine	1	39	1	0	36	3	25.00%	100.00%	92.50%
Mephedrone	18	22	13	5	21	1	92.86%	80.77%	85.00%
4-MPD	0	40	0	0	40	0	NA	100.00%	100.00%
MEAP	2	38	2	0	37	1	66.67%	100.00%	97.50%
CMC	0	40	0	0	40	0	NA	100.00%	100.00%
Methylone	13	27	2	11	27	0	100.00%	71.05%	72.50%
Ephylone	2	38	1	1	37	1	50.00%	97.37%	95.00%
Eutylone	14	26	14	0	25	1	93.33%	100.00%	97.50%
3,4-MDPHP	0	40	0	0	40	0	NA	100.00%	100.00%
Amphetamine	9	31	8	1	28	3	72.73%	96.55%	90.00%
Methamphetamine	16	24	12	4	19	5	70.59%	82.61%	77.50%
6-Acetylmorphine	0	40	0	0	39	1	0.00%	100.00%	97.50%
MDA	4	36	0	4	36	0	NA	90.00%	90.00%
MDMA	2	38	0	2	37	1	0.00%	94.87%	92.50%
DMA	16	24	5	11	23	1	83.33%	67.65%	70.00%
PMEA	0	40	0	0	40	0	NA	100.00%	100.00%
PMA	2	38	2	0	38	0	100.00%	100.00%	100.00%
PMMA	10	30	1	9	30	0	100.00%	76.92%	77.50%

a TP: true positive; FP: false positive; TN: true negative; FN: false negative; PPA: positive percent agreement (TP/PositivePredict); NPA: negative percent agreement (TN/NegativePredict); OPA: overall percent agreement (TP+ TN/Predict results).

The discordant results between the pDART screen and LC-QqQ-MS were reported in the 40 samples due to the difference in sensitivity between the two methods. The pDart-MS struggles to effectively resolve the quantification of low-concentration results obtained by LC-QqQ-MS. For instance, in the screening result of deschloroketamine, three false negatives detected by the LC-QqQ-MS assay were at concentrations below the pDART detection limit.

Qualitatively, the rates of agreement for positive and negative results can be used to measure a method’s positive percent agreement (PPA), negative percent agreement (NPA), and overall percent agreement (OPA). The PPA of methoxetamine, 4-MPD, CMC, 3,4-MDPHP, and PMEA could not be obtained due to the lack of positive specimens within the study. However, the NPA was 100%. The OPA of the pDART platform ranged from 70.0% to 100.0% for all evaluated drugs.

The quantitative results obtained by the pDART screening platform were compared to the LC-QqQ-MS confirmation results using weighted least-squares regression. The correlations of the two methods were assessed with a Pearson’s correlation coefficient. While the analysis and validation of the pDART method were established on the urine-based simulation, the results of the pDART screening test were significantly different from the LC–MS/MS quantitative result.^27^ The correlation coefficients of individual compounds ranged from 0.71 to 1 (Table S4).

**Figure 3:**
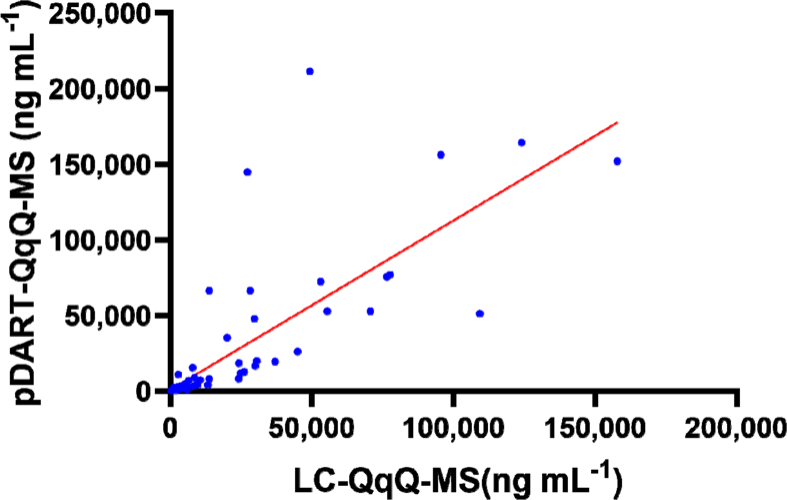
Correlation plot between concentrations measured by LC-QqQ-MS and pDART-QqQ-MS methods for 40 urine samples.

The difference might be caused by the slight amount of methanol from standards. It is important to note that the pDART method was developed as a rapid screening platform, whereas the UPLC method was developed for quantitative confirmation. The overall weighted kappa coefficient between pDART-QqQ-MS and LC-QqQ-MS reached 0.8152, indicating a positive correlation when the data was separated into six concentration levels (Figure 3), based on Fleiss-Cohen weights also including the LLOQ, and control level of amphetamines (Table S5).^28^

### Screening Nontargeted NPSs through Precursor Ion Scanning Approach

However, the development of NPSs exhibits rapid progression. Nontargeted screening via the QTRAP system may offer a solution to identify new drug abuses. The precursor ion (PI) scanning mode is valuable for investigating groups of compounds that generate common product ions after fragmentation in complex matrices. This approach is based on the automatic structural analysis using the hybrid triple quadrupole linear ion trap (LIT) technology of the QTRAP system.^29^ The information-dependent acquisition-enhanced product ion (IDA-EPI) scan was performed, involving precursor ion screening experiments of the product ions of interest with the acquisition of the product ion spectrum of these selective precursor ions in urine. The selection of product ions was based on the research of fragmentation pathways on emerging synthetic cathinone derivatives.^30,31^ We indented to conduct a nontargeted screening using this platform. For instance, the product ion of *m*/*z* 135 was chosen to detect the methylenedioxy moiety. Methylenedioxy-containing synthetic cathinones like dibutylone and MDPV were successfully detected under the paper-loaded screening method without preseparation of chromatography. Similarly, pyrrolidine ring-containing synthetic cathinones such as α-PVP and 4-chloro-α-PVP were detected under the PI scan for the pyrrolidine moiety (*m*/*z* 70). However, MDPV could not be detected under scanning for *m*/*z* 70 but *m*/*z* 125 (n-butylidenepyrrolidinium moiety) due to the messy result on low mass scanning in the PI mode. For more detailed experimental parameters and spectra, please refer to Table S6.

## Conclusion

The rapid semiquantitative method for analyzing 21 traditional drugs of abuse and NPSs in urine was conducted using direct analysis by pDART-QqQ-MS and was also the first to integrate for drug analysis. A triple quadrupole mass spectrometer in the MRM mode demonstrated sufficient selectivity to analyze these compounds simultaneously without chromatographic separation. The performance of the methodology was obtained and assessed through a validation protocol following recommendations from SWGTOX. The present method allows for low values of the LLOQ for synthetic cathinones and ketamines in urine. Although the sensitivity of amphetamine is higher than other analytes, the validation performance at the 400 ng mL^–1^ concentration level is still much lower than the cutoff value of currently immune-based devices for multiple drugs abused (1000 ng mL^–1^)^32,33^ and the reported threshold of regulatory enforcement (500 ngmL^–1^).^34^ In a study of 40 toxicological cases, pDART and LC–MS/MS had comparable detection rates, with an overall agreement of 70–100%. The rapid screening process of pDART analysis has a much shorter total processing time compared with LC–MS/MS (30 s vs 16 min). While a larger number of specimens is needed to assess the applicability of pDART-QqQ-MS, these results indicate that the method shows good promise as a drug screening method. On the other hand, pDART-MS has a quicker method development process in contrast to immune-based screening kits.^35^

On the other hand, the PI-IDA-EPI scanning methods have the potential to serve as a prescreening method to detect existing illegal drugs or their analogues in complex matrices. For the future application of the PI-IDA-EPI scanning method, the library of designer drug samples will need to be expanded to explore the database of the approach. Nevertheless, this semiquantitative pDART-QqQ-MS analysis allows for simple, rapid screening of multiple analytes within 30 s and with a limited volume of specimens. This study provides a potential utilization for future forensic preliminary screening.

## Supporting Information Available

Validation of forensic toxicological methods, TIC of MRM for samples, MRM of synthetic cathinones, MRM transitions and experimental parameters of analytes and internal standards, validation results, linear parameters, LOD, LC-QqQ-MS and pDART-QqQ-MS methods of 40 real urine samples, comparison of quantitative results of pDART screen and LC–MS/MS confirmation, overall weighted kappa coefficient between pDART-QqQ-MS and LC-QqQ-MS with data at six concentration levels, and PI-IDA-EPI experimental parameters (PDF)

## References

[ref1] Sajwani H. S. The dilemma of new psychoactive substances: A growing threat. Saudi Pharm. J. 2023, 31 (3), 348–350. 10.1016/j.jsps.2023.01.002. 37026049 PMC10071313

[ref2] Meyer M. R.; Maurer H. H. Review: LC coupled to low- and high-resolution mass spectrometry for new psychoactive substance screening in biological matrices - Where do we stand today?. Anal. Chim. Acta 2016, 927, 13–20. 10.1016/j.aca.2016.04.046. 27237833

[ref3] Pasin D.; Cawley A.; Bidny S.; Fu S. Current applications of high-resolution mass spectrometry for the analysis of new psychoactive substances: a critical review. Anal. Bioanal. Chem. 2017, 409 (25), 5821–5836. 10.1007/s00216-017-0441-4. 28634759

[ref4] Cheng K.-W.; Hsieh C.-M.; Chen H.-W.; Chi P.-C.; Yang D.-P.; Chan S.-H.; Chen J.-Y.; Hwa H.-L.; Fang C.-C.; Weng T.-I; Chen P.-S.; et al. Determination of synthetic cathinone alpha-pyrrolidinovalero-phenone and its metabolite in urine using solid-phase extraction and gas chromatography-mass spectrometry. Rapid Commun. Mass Spectrom. 2020, 34, e8579 10.1002/rcm.8579. 31502287

[ref5] Burd E. M. Validation of laboratory-developed molecular assays for infectious diseases. Clin. Microbiol. Rev. 2010, 23 (3), 550–576. 10.1128/CMR.00074-09. 20610823 PMC2901657

[ref6] de Araujo W. R.; Cardoso T. M. G.; da Rocha R. G.; Santana M. H. P.; Munoz R. A. A.; Richter E. M.; Paixao T.; Coltro W. K. T. Portable analytical platforms for forensic chemistry: A review. Anal. Chim. Acta 2018, 1034, 1–21. 10.1016/j.aca.2018.06.014. 30193622

[ref7] Boronat Ena M. d. M.; Cowan D. A.; Abbate V. Ambient ionization mass spectrometry applied to new psychoactive substance analysis. Mass Spectrom. Rev. 2023, 42 (1), 3–34. 10.1002/mas.21695. 34036620

[ref8] Kuo T. H.; Dutkiewicz E. P.; Pei J.; Hsu C. C. Ambient Ionization Mass Spectrometry Today and Tomorrow: Embracing Challenges and Opportunities. Anal. Chem. 2020, 92 (3), 2353–2363. 10.1021/acs.analchem.9b05454. 31825205

[ref9] Bianchi F.; Agazzi S.; Riboni N.; Erdal N.; Hakkarainen M.; Ilag L. L.; Anzillotti L.; Andreoli R.; Marezza F.; Moroni F.; et al. Novel sample-substrates for the determination of new psychoactive substances in oral fluid by desorption electrospray ionization-high resolution mass spectrometry. Talanta 2019, 202, 136–144. 10.1016/j.talanta.2019.04.057. 31171161

[ref10] McKenna J.; Jett R.; Shanks K.; Manicke N. E. Toxicological Drug Screening using Paper Spray High-Resolution Tandem Mass Spectrometry (HR-MS/MS). J. Anal. Toxicol. 2018, 42 (5), 300–310. 10.1093/jat/bky001. 29377996

[ref11] Morato N. M.; Pirro V.; Fedick P. W.; Cooks R. G. Quantitative Swab Touch Spray Mass Spectrometry for Oral Fluid Drug Testing. Anal. Chem. 2019, 91 (11), 7450–7457. 10.1021/acs.analchem.9b01637. 31074613

[ref12] Cody R. B.; Laramée J. A.; Durst H. D. Versatile New Ion Source for the Analysis of Materials in Open Air under Ambient Conditions. Anal. Chem. 2005, 77 (8), 2297–2302. 10.1021/ac050162j. 15828760

[ref13] Sisco E.; Forbes T. P. Forensic applications of DART-MS: A review of recent literature. Forensic Chem. 2021, 22, 100294 10.1016/j.forc.2020.100294. PMC9791994 36575658

[ref14] Gwak S.; Almirall J. R. Rapid screening of 35 new psychoactive substances by ion mobility spectrometry (IMS) and direct analysis in real time (DART) coupled to quadrupole time-of-flight mass spectrometry (QTOF-MS). Drug Test. Anal. 2015, 7 (10), 884–893. 10.1002/dta.1783. 25800348

[ref15] Zhou M.; McDonald J. F.; Fernandez F. M. Optimization of a direct analysis in real time/time-of-flight mass spectrometry method for rapid serum metabolomic fingerprinting. J. Am. Soc. Mass Spectrom. 2010, 21 (1), 68–75. 10.1016/j.jasms.2009.09.004. 19819164

[ref16] Zhang Y.; Zhang W.; Xin G.; Liu L.; Duan X.; Liu C. Rapid screening of nine illicit drugs in human blood and urine by direct analysis in real-time mass spectrometry. J. Forensic Sci. Med. 2019, 5 (3), 136 10.4103/jfsm.jfsm_30_18.

[ref17] Vasiljevic T.; Pawliszyn J. Direct analysis in real time (DART) and solid-phase microextraction (SPME) transmission mode (TM): a suitable platform for analysis of prohibited substances in small volumes. Anal. Methods 2019, 11 (30), 3882–3889. 10.1039/C9AY00797K.

[ref18] Vasiljevic T.; Gomez-Rios G. A.; Li F.; Liang P.; Pawliszyn J. High-throughput quantification of drugs of abuse in biofluids via 96-solid-phase microextraction-transmission mode and direct analysis in real time mass spectrometry. Rapid Commun. Mass Spectrom. 2019, 33 (18), 1423–1433. 10.1002/rcm.8477. 31063263

[ref19] Weng C. Y.; Kuo T. H.; Chai L. M. X.; Zou H. B.; Feng T. H.; Huang Y. J.; Tsai J. C.; Wu P. H.; Chiu Y. W.; Lan E. I.; et al. Rapid Quantification of Gut Microbial Short-Chain Fatty Acids by pDART-MS. Anal. Chem. 2020, 92 (22), 14892–14897. 10.1021/acs.analchem.0c03862. 33151059

[ref20] Hsieh H. Y.; Li L. H.; Hsu R. Y.; Kao W. F.; Huang Y. C.; Hsu C. C. Quantification of Endogenous Cholesterol in Human Serum on Paper Using Direct Analysis in Real Time Mass Spectrometry. Anal. Chem. 2017, 89 (11), 6146–6152. 10.1021/acs.analchem.7b00943. 28505411

[ref21] Scientific Working Group for Forensic Toxicology (SWGTOX) standard practices for method validation in forensic toxicology. J. Anal. Toxicol. 2013, 37 (7), 452–474. 10.1093/jat/bkt054. 23934984

[ref22] Chen J. Y.; Chen G. Y.; Wang S. Y.; Fang C. C.; Chen L. Y.; Weng T. I. Development of an analytical method to detect simultaneously 219 new psychoactive substances and 65 other substances in urine specimens using LC-QqQ MS/MS with CriticalPairFinder and TransitionFinder. Talanta 2022, 238, 122979 10.1016/j.talanta.2021.122979. 34857319

[ref23] Haunschmidt M.; Klampfl C. W.; Buchberger W.; Hertsens R. Rapid identification of stabilisers in polypropylene using time-of-flight mass spectrometry and DART as ion source. Analyst 2010, 135 (1), 80–85. 10.1039/B911040B. 20024185

[ref24] Frey B. S.; Damon D. E.; Badu-Tawiah A. K. Emerging trends in paper spray mass spectrometry: Microsampling, storage, direct analysis, and applications. Mass Spectrom. Rev. 2020, 39 (4), 336–370. 10.1002/mas.21601. 31491055 PMC7875099

[ref25] Edelbroek P. M.; Heijden J. v. d.; Stolk L. M. L. Dried Blood Spot Methods in Therapeutic Drug Monitoring: Methods, Assays, and Pitfalls. Ther. Drug Monit. 2009, 31 (3), 327–336. 10.1097/FTD.0b013e31819e91ce. 19349929

[ref26] Gundersen P. O. M.; Spigset O.; Josefsson M. Screening, quantification, and confirmation of synthetic cannabinoid metabolites in urine by UHPLC-QTOF-MS. Drug Test. Anal. 2019, 11 (1), 51–67. 10.1002/dta.2464. 29996011 PMC6585856

[ref27] Pum J. A practical guide to validation and verification of analytical methods in the clinical laboratory. Adv. Clin. Chem. 2019, 90, 215–281. 10.1016/bs.acc.2019.01.006. 31122610

[ref28] Tang W.; Hu J.; Zhang H.; Wu P.; He H. Kappa coefficient: a popular measure of rater agreement. Shanghai Arch. Psychiatry 2015, 27 (1), 62–67. 10.11919/j.issn.1002-0829.215010. 25852260 PMC4372765

[ref29] Lee H.-m.; Lee B. J. A novel approach to simultaneous screening and confirmation of regulated pharmaceutical compounds in dietary supplements by LC/MS/MS with an information-dependent acquisition method. Food Addit. Contam. - Chem. Anal. Control Expo. Risk Assess. 2011, 28 (4), 396–407. 10.1080/19440049.2011.551947. 21416414

[ref30] Fowble K. L.; Shepard J. R. E.; Musah R. A. Identification and classification of cathinone unknowns by statistical analysis processing of direct analysis in real time-high resolution mass spectrometry-derived ″neutral loss″ spectra. Talanta 2018, 179, 546–553. 10.1016/j.talanta.2017.11.020. 29310273

[ref31] Davidson J. T.; Sasiene Z. J.; Jackson G. P. Fragmentation pathways of odd- and even-electron N-alkylated synthetic cathinones. Int. J. Mass Spectrom. 2020, 453, 116354 10.1016/j.ijms.2020.116354.

[ref32] Poklis A.; Still J.; Slattum P. W.; Edinboro L. F.; Saady J. J.; Costantino A. Urinary Excretion of d-Amphetamine Following Oral Doses in Humans: Implications for Urine Drug Testing. J. Anal. Toxicol. 1998, 22 (6), 481–486. 10.1093/jat/22.6.481. 9788523

[ref33] Moeller K. E.; Lee K. C.; Kissack J. C. Urine Drug Screening: Practical Guide for Clinicians. Mayo Clin. Proc. 2008, 83 (1), 66–76. 10.4065/83.1.66. 18174009

[ref34] Kulig K. Interpretation of Workplace Tests for Cannabinoids. J. Med. Toxicol. 2017, 13 (1), 106–110. 10.1007/s13181-016-0587-z. 27686239 PMC5330962

[ref35] Picotti P.; Aebersold R. Selected reaction monitoring-based proteomics: workflows, potential, pitfalls and future directions. Nat. Methods 2012, 9 (6), 555–566. 10.1038/nmeth.2015. 22669653

