# Supplementary material for: Rapid Screening of New Psychoactive Substances Using pDART-QqQ-MS
Source: J Am Soc Mass Spectrom. 2024 Apr 23;35(6):1370–6. doi: 10.1021/jasms.4c00124 (PMC11157655; doi:10.1021/jasms.4c00124)

Supplementary Information
For
Rapid Screening of New Psychoactive Substances Using pDART-QqQ-MS

Wei-Hsin Hsu[a‡], Kai-Wen Cheng[a‡], Tzu-Hsuan Feng[a], Ju-Yu Chen[b], Guan-Yuan Chen[b,c], Lian-Yu Chen[d,e], Te-I Weng[b,c*], Cheng-Chih Hsu[a,f*]

[a] Department of Chemistry, National Taiwan University, Taipei 10617, Taiwan

[b] Forensic and Clinical Toxicology Center National Taiwan University College of Medicine and National Taiwan University Hospital, Taipei 10051, Taiwan

[c] Department and Graduate Institute of Forensic Medicine, College of Medicine, National Taiwan University, Taipei 10051, Taiwan

[d] Institute of Epidemiology and Preventive Medicine, National Taiwan University, Taipei 10051, Taiwan

[e] Kunming Prevention and Control Center, Taipei City Hospital, Taipei 108203, Taiwan.

[f] Leeuwenhoek Laboratories Co. Ltd., No. 71, Fanglan Rd, Taipei, 106038, Taiwan.

Table of Contents

Experimental Procedures

Validation of Forensic Toxicological Methods 2

Supplement Figures and Tables

Figure S1. The total ion chromatogram (TIC) of MRM for air, paper, and matrix blank samples, with and without internal standard. 3

Figure S2. The MRM of synthetic cathinones for matrix blank, methanol, 5 ppb, and 10 ppb. 4

Supplementary Table S1. MRM transitions and experimental parameters for all analytes and internal standards 5

Supplementary Table S2. Validation results, linear parameters, LOD, and other validation parameters were assessed at LLOQ, 400, and 750 ngmL-1 levels. 9

Supplementary Table S3. The results from 40 real urine samples of drug-abused subjects were analyzed using both LC-QqQ-MS and pDART-QqQ-MS methods. 10

Supplementary Table S4. Comparison of quantitative results of pDART screen and LC-MS/MS confirmation 14

Supplementary Table S5. The overall weighted Kappa coefficient between pDART-QqQ-MS and LC-QqQ-MS when the data was separated into six concentration levels 14

Supplementary Table S6. PI-IDA-EPI experimental parameters and Fragmentation of the analytes detected in precursor ion scan 15

Experimental Procedures

Validation of Forensic Toxicological Methods

Each calibration point was run in quintuplicate. The normalized area for each analyte was calculated using the selected internal standard signal area (Supplementary Table S1). Linear calibration curves were constructed using a least square regression model with the spiked concentration (1/x) reciprocal as a weighting factor. The calibration parameter coefficient of determination (R^2) was used to roughly estimate the linearity. The limit of detection (LOD) was estimated using the ratio of the average normalized area with 3.3 times the standard deviation of blanks within five measurements. The lower limit of qualification (LLOQ) was defined as the standard's concentration that produced a bias within ±20%. For all the analytes, interference, carryover effect, accuracy, system precision, repeatability, matrix effect, and inter-day precision were evaluated at several different concentrations, including LLOQ. Interference was evaluated using blank urine samples from three males and four females at different ionization temperatures. Accuracy, expressed as bias%, compared the concentration calculated from the linear model and the nominal concentration. Precision, shown as the coefficient of variation (%CV), assessed the level of variation at each concentration. System precision was used to assess the stability of the pDART system within five measurements by a single testing sample. Method precision revealed the variation within three independent preparations. Matrix effect, or ion suppression, was investigated by comparing the 50% methanol spiked with the analytes with blank spiked samples. Inter-day precision was used to evaluate the stability of the prepared solution

The carryover effect was assessed by measuring the double blank solution (pooled urine without any intentional addition of analytes and internal standard) in the same testing set after the highest concentration samples (1000 ngmL^{-1}) used in calibration curves. Stability was investigated through the inter-day precision and method bias analyzed with the same test solution stored at -20°C for 3 days in quintuplicate. Quality controls were analyzed in triplicate at the concentrations 20, 40, 75, 200, 400, 750 ngmL-1, to confirm the deflection of the linearity against the default linearity within the range of 0.8-1.2.

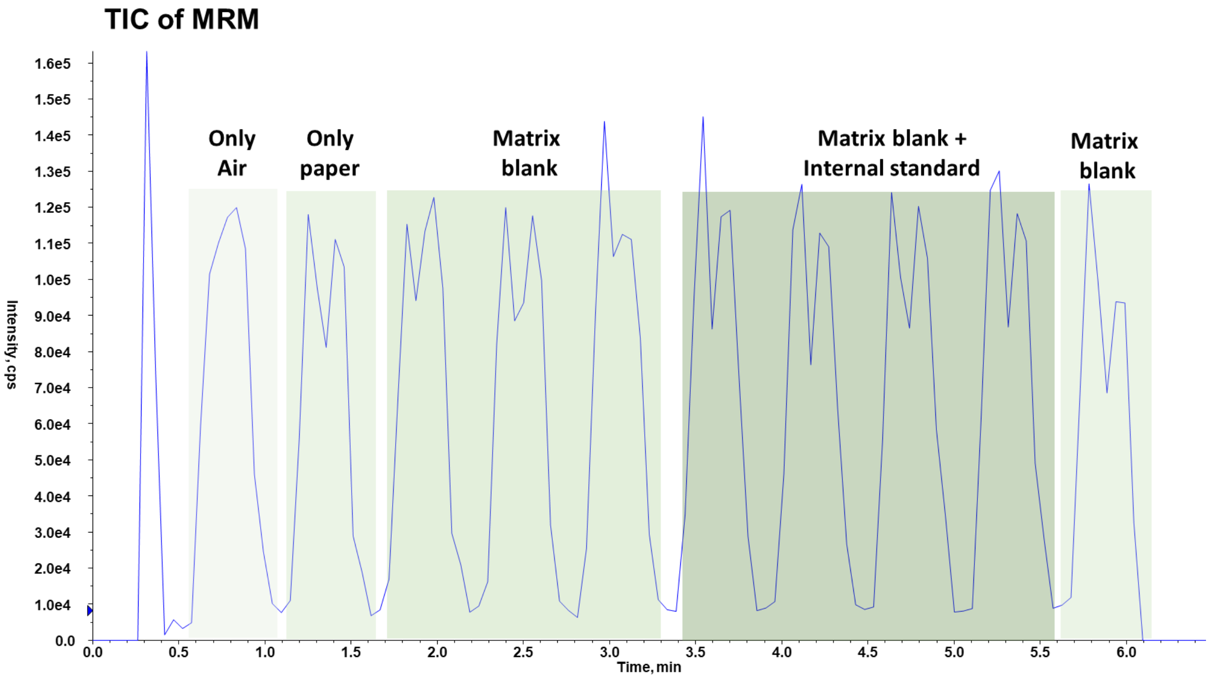

Figure S1. The total ion chromatogram (TIC) of MRM for air, paper, and matrix blank samples, with and without internal standard.

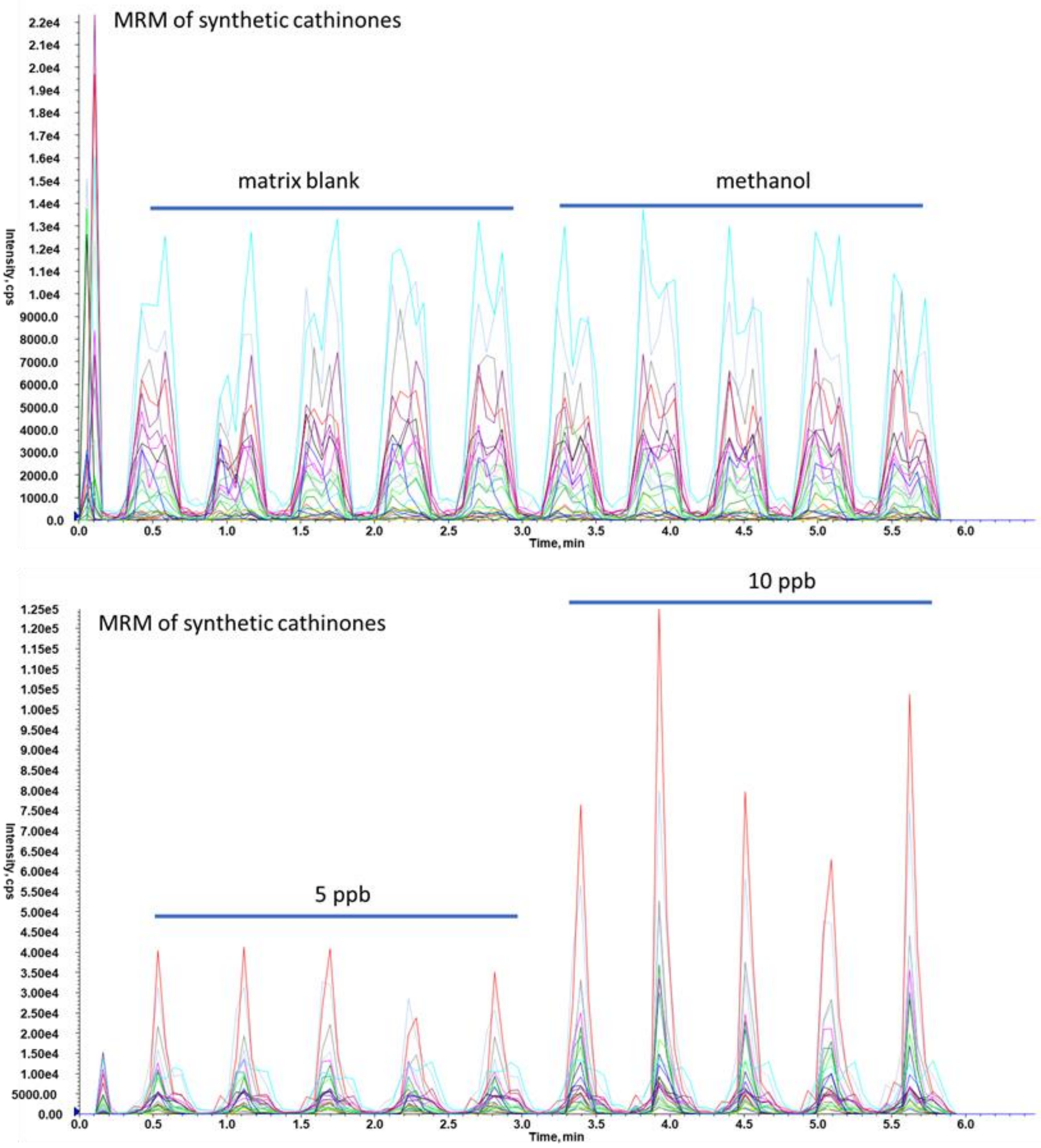

Figure S2. The MRM of synthetic cathinones for matrix blank, methanol, 5 ppb, and 10 ppb.

Supplementary Table S1. MRM transitions and experimental parameters for all analytes and internal standards

**Compound**		**Precursor ion [M + H]$^+$**	**DP**	**EP**	**Fragment**	***m/z***	**CE**	**CXP**	**Internal standard**	**Normalization fragment**	**Ionization helium temperature**
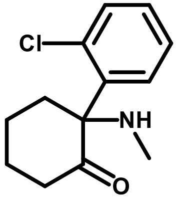	Ketamine	238	80	10	Quantifier	125	40	10	Ketamine-D_4	242/129	300
					Qualifier	220	22	10			
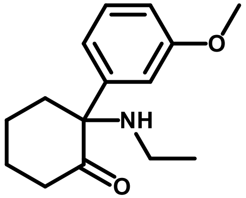	Methoxetamine	248	76	12	Quantifier	203	19	14	Ketamine-D_4	242/129	300
					Qualifier	121	39	8			
					Qualifier	175	25	12			
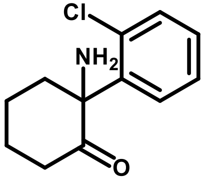	Norketamine	224	70	10	Quantifier	179	22	9	Ketamine-D_4	242/129	300
					Qualifier	125	30	12			
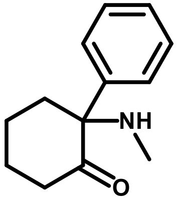	Deschloroketamine	204	65	10	Quantifier	173	19	12	Ketamine-D_4	242/129	300
					Qualifier	145	23	10			
					Qualifier	91	35	7			
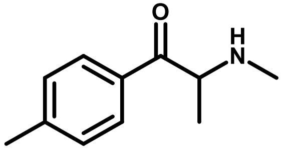	Mephedrone	178	80	10	Quantifier	145	18	10	Ketamine-D_4	242/129	300
					Qualifier	160	28	10			
					Qualifier	91	47	10			

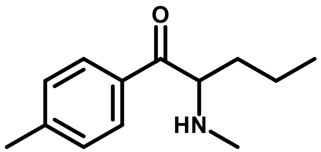	4-MPD	206	79	8	Quantifier	188	17	13	Ketamine-D_4	242/129	300
					Qualifier	145	30	10			
					Qualifier	105	28	7			
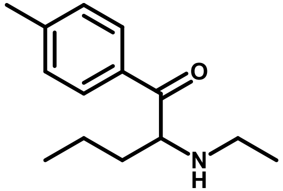	MEAP	220	80	12	Quantifier	202	19	14	Ketamine-D_4	242/129	300
					Qualifier	105	31	7			
					Qualifier	160	25	11			
					Qualifier	175	20	12			
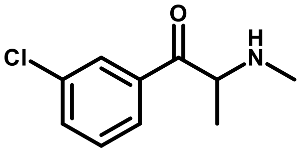	CMC	198	80	9	Quantifier	180	16	12	Ketamine-D_4	242/129	300
					Qualifier	145	26	10			
					Qualifier	144	43	10			
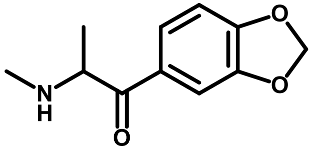	Methylone	208	80	10	Quantifier	132	37	10	Ketamine-D_4	242/129	300
					Qualifier	160	24	10			
					Qualifier	190	17	10			
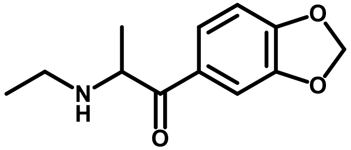	Ephylone	250	72	11	Quantifier	202	25	14	Ketamine-D_4	242/129	300
					Qualifier	232	20	9			
					Qualifier	189	33	13			
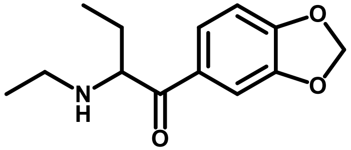	Eutylone	236	70	9	Quantifier	160	35	11	Ketamine-D_4	242/129	300
					Qualifier	218	20	10			
					Qualifier	188	25	13			

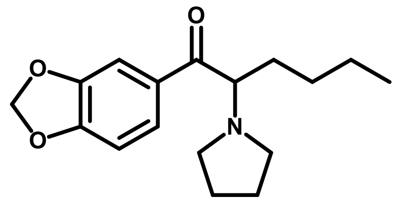	3,4-MDPHP	290	102	7	Quantifier	140	36	10	Ketamine-D4	242/129	300
					Qualifier	219	25	15			
					Qualifier	189	30	14			
					Qualifier	135	35	10			
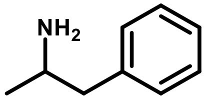	Amphetamine	136	50	10	Quantifier	91	21	10	Ketamine-D4	242/129	300
					Qualifier	119	11	10			
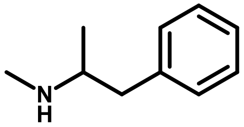	Methamphetamine	150	80	10	Quantifier	119	13	10	Methamphetamine-D8	158/93	300
					Qualifier	91	30	10			
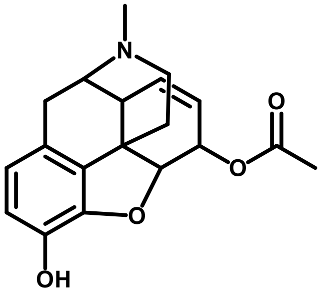	6-acetylmorphine	328	138	9	Quantifier	211	35	15	Methamphetamine-D8	158/93	400
					Qualifier	193	37	14			
					Qualifier	165	51	11			
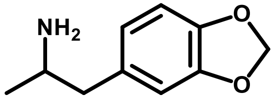	MDA	180	55	10	Quantifier	133	24	9	Methamphetamine-D8	158/93	400
					Qualifier	135	25	10			
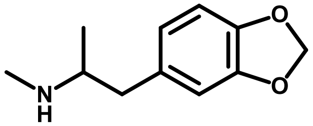	MDMA	194	65	12	Quantifier	163	16	12	Ketamine-D4	242/129	300
					Qualifier	133	27	10			
					Qualifier	135	28	10			

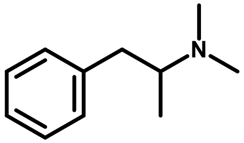	DMA	164	70	11	Quantifier	91	27	6	Ketamine-D_4	242/129	300
					Qualifier	119	17	8			
					Qualifier	65	56	8			
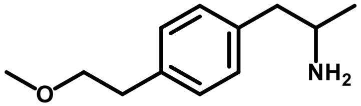	PMEA	194	66	9	Quantifier	121	30	9	Ketamine-D_4	242/129	300
					Qualifier	149	17	11			
					Qualifier	91	46	10			
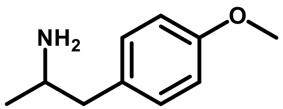	PMA	166	53	7	Quantifier	121	26	10	Ketamine-D_4	242/129	300
					Qualifier	91	40	8			
					Qualifier	78	53	10			
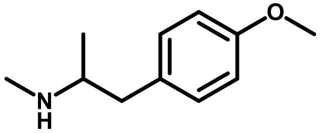	PMMA	180	56	13	Quantifier	149	16	10	Ketamine-D_4	242/129	300
					Qualifier	91	42	9			
					Qualifier	78	53	10			
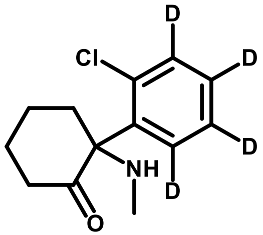	Ketamine-D_4	242	80	10		129	38	10	Internal standards		300
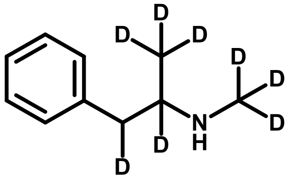	Methamphetamine-D_8	158	62	8		93	18	7			400
						124	35	9			

Supplementary Table S2. Validation results, linear parameters, LOD, and other validation parameters were assessed at LLOQ, 400, and 750 ngmL^{-1} levels.

Compound	Linearity					LLOQ							QC 400 ngmL^{-1}						QC 750 ngmL^{-1}					
	Range	Slope	Intercept	R square	Cal LOD (ngmL^{-1})	Conc. (ngmL^{-1})	System precision (%CV)	Method precision (%CV)	Accuracy (%Bias)	Matrix Effect (%)	Inter-day precision (%CV)	Stability (%Bias)	System precision (%CV)	Method precision (%CV)	Accuracy (%Bias)	Matrix Effect (%)	Inter-day precision (%CV)	Stability (%Bias)	System precision (%CV)	Method precision (%CV)	Accuracy (%Bias)	Matrix Effect (%)	Inter-day precision (%CV)	Stability (%Bias)
Ketamine	5-1000	8.86E-03	4.63E-03	0.9916	2.00	20	2.65	6.31	1.72	116.75	10.96	11.63	2.77	15.71	1.35	105.47	18.27	4.64	3.52	7.94	1.43	89.97	6.06	-2.01
Methoxetamine	1-1000	1.53E-02	2.16E-03	0.9912	3.06	20	4.62	7.85	-1.40	106.05	8.63	7.38	3.18	15.28	0.75	115.76	21.14	20.36	5.82	7.92	0.84	101.77	11.51	11.35
Norketamine	1-1000	6.88E-03	1.78E-03	0.9938	3.92	50	5.69	12.76	-3.86	139.32	10.44	3.96	4.83	12.44	-0.30	145.55	17.43	13.81	7.18	7.89	1.24	113.36	8.55	-6.74
Deschloroketamine	1-1000	7.83E-03	1.09E-03	0.9916	2.44	50	3.77	13.07	-3.17	87.53	10.81	-6.03	9.56	-3.34	20.17	86.69	11.92	6.21	4.71	7.12	3.84	65.92	11.11	-10.63
Mephedrone	10-1000	3.44E-03	1.70E-03	0.9867	6.43	20	6.87	16.40	-1.8 7	42.44	12.39	6.12	5.15	10.42	-0.01	39.26	12.29	-1.57	4.34	9.83	1.14	49.11	10.02	7.04
4-MPD	10-1000	5.97E-02	7.56E-02	0.9874	8.91	20	12.56	16.88	12.16	105.05	5.52	-7.57	13.41	16.98	-5.89	73.60	19.84	-5.94	9.71	17.40	-1.39	86.78	19.71	-11.04
MEAP	5-1000	7.64E-02	4.07E-02	0.9923	3.97	20	5.21	9.06	0.58	103.45	8.58	-4.72	9.35	12.81	2.76	77.33	18.02	-8.65	8.14	15.72	3.51	90.75	14.45	-9.83
CMC	10-1000	4.67E-03	-2.68E-03	0.9929	9.52	20	7.86	18.98	-4.20	43.13	16.77	10.13	7.03	13.68	-2.78	44.96	18.80	15.98	6.95	14.70	-1.12	56.43	7.15	13.38
Methylone	10-1000	1.53E-03	2.77E-03	0.9828	28.75	50	5.35	10.54	-0.69	49.45	17.01	10.41	4.56	10.66	1.99	35.27	12.97	8.81	6.51	12.20	5.68	46.46	11.23	-3.55
Ephylone	1-1000	1.46E-02	3.23E-04	0.9913	0.38	20	6.64	16.41	-3.32	63.77	9.31	1.62	10.05	18.05	-8.55	54.03	18.69	-8.94	12.88	19.02	1.39	65.32	19.02	-14.11
Eutylone	10-1000	1.53E-03	1.85E-03	0.9862	11.81	20	5.44	16.94	7.13	43.86	13.81	-2.43	10.24	18.56	5.96	42.62	20.84	-5.46	5.35	16.05	-7.47	44.00	15.25	-4.97
3,4-MDPHP	1-1000	6.56E-03	-3.23E-04	0.9830	0.42	20	4.14	13.15	-4.50	96.76	11.38	0.06	10.81	26.07	-6.52	64.47	22.86	13.16	6.00	24.11	-10.57	76.88	19.63	-21.56
Amphetamine	10-1000	7.41E-03	8.84E-02	0.8343	113.33	75	10.14	18.36	54.38	143.61	25.70	-17.07	5.41	10.91	2.39	121.03	12.89	11.74	3.49	19.55	-7.08	120.00	27.32	-5.53
Methamphetamine	5-1000	3.68E-03	1.02E-03	0.9909	5.94	20	3.08	14.39	-0.65	122.20	15.23	9.79	8.02	11.34	3.39	107.80	13.19	-0.85	10.80	18.36	4.37	107.08	12.50	-5.21
6-acetylmorphine	10-1000	2.48E-04	1.56E-03	0.9321	66.39	50	17.80	28.39	19.62	136.49	23.96	2.83	20.83	25.07	0.36	109.42	23.61	-2.73	15.36	21.11	-8.08	71.52	32.72	10.30
MDA	10-1000	8.92E-04	3.05E-03	0.9822	21.46	50	10.51	21.99	0.15	102.18	10.75	2.76	8.46	16.43	5.27	114.31	18.63	1.12	13.06	14.89	-6.91	65.62	25.32	-19.25
MDMA	5-1000	2.07E-03	1.08E-05	0.9958	3.21	20	7.79	15.26	4.23	52.17	11.53	12.94	8.29	8.85	-0.48	53.30	17.18	-8.26	13.71	16.39	7.08	47.61	29.30	8.42
DMA	10-1000	1.24E-02	2.85E-02	0.9836	25.26	50	4.07	14.12	4.19	101.47	6.66	-0.94	8.06	12.79	-4.08	94.46	8.14	14.17	5.96	10.54	-2.59	102.36	7.22	4.14
PMEA	10-1000	7.21E-03	2.59E-02	0.9781	25.84	50	9.64	12.04	14.66	126.83	14.54	4.90	7.93	9.19	-3.90	95.33	9.82	12.77	6.11	9.14	-2.05	103.73	9.29	13.04
PMA	10-1000	3.64E-03	1.09E-02	0.9822	38.83	50	14.08	17.56	8.76	136.59	15.52	14.85	2.98	13.42	-6.19	97.28	13.85	-17.60	7.30	13.09	-3.39	111.45	8.26	5.06
PMMA	1-1000	6.32E-03	7.12E-04	0.9963	2.41	20	3.76	14.44	0.15	99.57	16.01	-1.90	5.47	12.87	1.45	90.20	12.41	21.71	13.38	17.18	-5.55	97.36	9.68	7.32

Supplementary Table S3. The results from 40 real urine samples of drug-abused subjects were analyzed using both LC-QqQ-MS and pDART-QqQ-MS methods.

**Compound**	**LC-QqQ-MS (ngmL^{-1})**	**pDART-QqQ-MS (ngmL^{-1})**	**No.**	**pDART-QqQ-MS area ratio (Area$_{Analyte}$/Area$_{Internal\ standard}$)**					
				**Triple**			**Mean**	**SD**	**CV%**
Ketamine	2784.9	772.54	#01	7.04	7.13	6.35	6.84	0.06	0.91
	3130.9	1244.35	#03	10.63	10.55	11.86	11.01	0.07	0.61
	9404.2	3897.80	#04	35.47	34.26	33.71	34.48	0.03	0.08
	343.3	140.70	#08	1.23	1.32	1.20	1.25	0.05	4.07
	1526.1	592.73	#10	4.88	5.46	5.41	5.25	0.06	1.15
	710.2	279.69	#11	2.45	2.51	2.49	2.48	0.01	0.49
	70.8	50.77	#14	0.53	0.49	0.35	0.46	0.22	47.29
	1427.7	633.85	#15	5.57	5.96	5.31	5.61	0.06	1.05
	6050.7	2110.28	#18	24.32	14.69	17.00	18.67	0.27	1.44
	914.2	506.71	#21	4.43	4.25	3.46	4.04	0.13	3.13
	1154.7	1056.36	#28	8.53	9.13	7.61	8.42	0.09	1.08
	35.7	33.74	#29	0.29	0.32	0.22	0.28	0.17	62.57
	1019.2	566.94	#31	5.26	4.26	4.06	4.52	0.14	3.15
	10516.9	7488.92	#32	60.22	69.16	49.67	59.68	0.16	0.27
	39.5	25.27	#36	0.18	0.29	0.16	0.21	0.34	160.90
	439.9	277.06	#39	2.43	1.83	2.39	2.21	0.15	6.89
Norketamine	844.8	306.35	#01	2.26	2.34	1.73	2.11	0.16	7.50
	4334.3	2601.00	#03	16.43	20.25	16.88	17.85	0.12	0.65
	8112	3190.60	#04	22.07	20.96	22.67	21.90	0.04	0.18
	128.8	206.47	#08	1.43	1.47	1.36	1.42	0.04	2.73
	1598.7	568.06	#10	4.16	3.71	3.84	3.90	0.06	1.54
	438.7	314.44	#11	2.23	2.31	1.96	2.16	0.08	3.91
	100.9	69.99	#14	0.42	0.55	0.48	0.49	0.14	28.13
	263	188.38	#15	1.38	1.15	1.37	1.30	0.10	7.58
	6445.8	2725.27	#18	26.57	12.65	16.90	18.71	0.38	2.04
	933.6	512.90	#21	3.17	3.29	3.74	3.40	0.09	2.59
	772.8	1181.91	#28	9.11	7.40	6.96	7.82	0.15	1.86
	160.1	114.36	#29	0.69	0.94	0.67	0.77	0.20	25.98
	973.9	318.38	#31	1.79	2.75	1.81	2.12	0.26	12.22
	3858.9	3682.53	#32	21.56	24.65	26.86	24.36	0.11	0.45
	23.7	11.91	#33	0.09	0.09	0.09	0.09	0.03	28.33

	25	23.76	#35	0.16	0.17	0.17	0.17	0.02	13.49
	68.2	36.65	#36	0.25	0.29	0.22	0.25	0.15	58.91
	311.9	102.12	#39	0.58	0.79	0.69	0.69	0.16	22.98
Deschloroketamine	37.4	15.45	#03	0.11	0.12	0.15	0.13	0.18	146.79
	289.3	94.53	#04	0.72	0.76	0.75	0.74	0.03	3.99
	19.5	7.65	#08	0.07	0.06	0.07	0.06	0.03	49.56
	25.7	12.09	#11	0.10	0.11	0.09	0.10	0.06	62.03
Mephedrone	95536.3	156204.31	#01	556.72	550.31	506.33	537.79	0.05	0.01
	53108.8	72439.49	#02	251.64	253.18	243.38	249.40	0.02	0.01
	1072.5	954.03	#07	0.38	0.54	0.46	0.46	0.17	37.58
	100.9	133.22	#08	0.04	0.05	0.05	0.05	0.15	328.16
	37001.3	19555.64	#13	73.04	61.28	67.67	67.33	0.09	0.13
	141.6	274.47	#19	0.64	1.16	1.04	0.95	0.29	30.43
	55418.4	52924.94	#21	142.60	136.20	142.70	140.50	0.03	0.02
	13625.7	66475.70	#26	276.80	125.60	127.00	176.47	0.49	0.28
	435.8	463.80	#29	0.94	1.98	0.85	1.26	0.50	39.88
	27186.4	144954.55	#31	259.50	346.40	548.40	384.77	0.39	0.10
	37.9	727.02	#33	1.59	2.45	1.83	1.96	0.23	11.65
	24897.5	11929.82	#35	19.40	35.00	40.67	31.69	0.35	1.10
	39.8	62.18	#36	0.09	0.27	0.21	0.19	0.50	263.97
	49266.7	211565.50	#39	323.70	653.20	707.80	561.57	0.37	0.07
Methylone	112.4	107.71	#13	0.17	0.17	0.17	0.17	0.01	7.68
	2444.2	2714.90	#14	4.35	4.33	3.82	4.16	0.07	1.74
MEAP	1449.2	629.64	#01	47.01	50.52	46.86	48.13	0.04	0.09
	30485.4	19902.32	#02	2028.32	1349.69	1180.56	1519.52	0.30	0.02
	7.5	6.81	#08	0.46	0.72	0.55	0.58	0.23	40.45
Ephylone	16.5	27.70	#02	0.52	0.37	0.33	0.41	0.25	62.66
	8510.1	8896.85	#37	123.80	135.70	78.49	112.66	0.27	0.24
Eutylone	157909	152115.18	#01	260.29	196.37	240.93	232.53	0.14	0.06
	70680.6	52775.05	#02	108.92	76.23	56.88	80.67	0.33	0.40
	2099.1	562.13	#07	0.71	0.83	1.04	0.86	0.19	22.17
	5946.1	850.71	#08	1.15	1.41	1.35	1.30	0.10	7.94
	124155.2	164521.00	#09	261.15	258.32	235.00	251.49	0.06	0.02
	26110.3	12782.51	#14	17.15	22.13	19.35	19.54	0.13	0.65
	30.9	34.71	#19	0.05	0.06	0.06	0.05	0.15	273.08
	76488.1	75717.66	#21	94.99	94.24	60.96	83.40	0.23	0.28

	2820.9	11133.27	#23	11.66	13.26	11.88	12.27	0.07	0.58
	7838.1	15530.90	#26	17.69	17.23	16.41	17.11	0.04	0.22
	20027.9	35485.21	#31	41.27	31.60	44.39	39.09	0.17	0.44
	28142.2	66444.19	#33	75.68	83.83	60.04	73.18	0.17	0.23
	77645.7	77067.52	#34	107.80	71.84	75.01	84.88	0.23	0.28
	5347.2	1598.30	#35	1.10	2.23	1.96	1.77	0.33	18.94
	29718.9	47891.20	#39	57.80	44.02	56.43	52.75	0.14	0.27
Amphetamine	216.7	114.33	#05	1.10	1.09	0.62	0.94	0.30	31.71
	29980.9	16805.98	#06	126.17	118.72	128.99	124.63	0.04	0.03
	9353.9	4898.00	#12	30.19	38.95	40.02	36.38	0.15	0.41
	21.8	90.41	#16	1.00	0.85	0.43	0.76	0.39	51.85
	2503.2	753.34	#17	5.52	5.89	5.61	5.67	0.03	0.61
	1404.5	452.63	#20	3.59	3.10	3.64	3.44	0.09	2.50
	6434.5	7021.00	#22	44.70	61.38	65.19	57.09	0.19	0.33
	909.9	1230.81	#24	12.64	10.39	7.84	10.29	0.23	2.27
	4184.2	2040.88	#32	11.33	18.82	20.36	16.84	0.29	1.70
	112.1	101.00	#36	1.28	1.18	1.01	1.16	0.12	10.42
	584.1	308.06	#38	1.73	2.30	4.46	2.83	0.51	17.95
Methamphetamine	134.5	70.24	#01	0.41	0.55	0.38	0.45	0.21	46.03
	158.1	43.09	#05	0.31	0.38	0.19	0.29	0.34	115.08
	109309.4	51249.00	#06	338.51	264.79	270.58	291.29	0.14	0.05
	78.6	861.76	#09	4.37	6.99	4.35	5.23	0.29	5.54
	44932.9	26269.80	#12	164.25	242.13	166.43	190.93	0.23	0.12
	51.5	28.21	#16	0.16	0.18	0.24	0.19	0.22	114.47
	24070.4	8463.13	#17	45.89	56.66	49.39	50.65	0.11	0.21
	25.9	7.57	#19	0.04	0.05	0.10	0.06	0.55	925.07
	13200.8	4252.03	#20	21.53	31.14	27.88	26.85	0.18	0.68
	19.9	183.49	#21	3.04	1.15	2.21	2.13	0.45	20.90
	24121.4	18707.30	#22	132.10	226.40	229.60	196.03	0.28	0.14
	5222.3	4825.30	#24	51.64	54.89	45.63	50.72	0.09	0.18
	6.3	262.82	#25	2.21	1.85	4.82	2.96	0.55	18.50
	85.7	119.87	#29	1.11	2.17	1.12	1.46	0.42	28.44
	13669.1	8299.15	#32	68.72	82.13	110.40	87.08	0.24	0.28
	209.9	211.71	#34	2.25	2.49	2.54	2.43	0.06	2.65
	2443.3	1303.38	#36	17.30	13.15	11.11	13.85	0.23	1.64
	2	66.41	#37	1.27	0.95	0.50	0.91	0.43	47.22

	1658.9	813.84	#38	5.44	6.50	14.25	8.73	0.55	6.31
6-acetylmorphine	441.9	367.98	#30	0.13	0.08	0.05	0.09	0.46	537.32
MDMA	4.5	24.27803748	#25	0.04	0.02	0.04	0.03	0.35	1014.27
DMA	630.8	474.05	#06	3.95	3.96	4.62	4.18	0.09	2.23
	155.5	166.92	#12	1.48	1.40	1.60	1.49	0.07	4.37
	30.9	39.17	#17	0.38	0.37	0.39	0.38	0.04	9.27
	13.7	2.23	#20	0.16	0.15	0.14	0.15	0.07	46.41
	1265	2316.35	#22	15.26	20.56	16.06	17.29	0.17	0.96
	48.6	190.01	#32	1.37	1.37	1.65	1.46	0.11	7.53
PMA	2716.9	1490.70	#13	4.24	4.37	3.63	4.08	0.10	2.38
	132.1	168.72	#29	0.31	0.71	0.38	0.47	0.46	98.32
PMMA	654.6	647.76	#29	1.64	4.20	2.12	2.65	0.51	19.38

Supplementary Table S4. Comparison of quantitative results of pDART screen and LC-MS/MS confirmation

**Compound**	**Number**	**Correlation coefficient**	**Equation**	**Slope (95%CI)**	**Intercept (95%CI)**
Ketamine	16	0.937	Y = 0.5497*X - 129.5	0.4327 to 0.6667	-602.7 to 343.6
Norketamine	18	0.903	Y = 0.4610*X + 144.7	0.3449 to 0.5770	-187.2 to 476.7
Deschloroketamine	4	1.000	Y = 0.3166*X + 2.991	0.2912 to 0.3421	-0.7427 to 6.725
Mephedrone	14	0.712	Y = 1.705*X + 9191	0.6483 to 2.761	-31088 to 49470
MEAP	3	1.000	Y = 0.6577*X - 156.9	0.5374 to 0.7781	-2277 to 1964
Eutylone	15	0.949	Y = 1.029*X + 4069	0.8235 to 1.235	-8954 to 17091
Amphetamine	11	0.976	Y = 0.5643*X + 216.5	0.4691 to 0.6595	-715.4 to 1148
Methamphetamine	19	0.985	Y = 0.4835*X + 541.1	0.4399 to 0.5271	-705.6 to 1788
DMA	6	0.952	Y = 1.688*X - 71.91	0.9336 to 2.443	-510.3 to 366.5
Overall	59	0.807	Y = 1.663*X + 731.0	1.253 to 2.073	-6676 to 8138

Supplementary Table S5. The overall weighted Kappa coefficient between pDART-QqQ-MS and LC-QqQ-MS when the data was separated into six concentration levels

**LC-MS/MS Quantitative result ($ngmL^{-1}$)**	**pDART-QqQ-MS Quantitative result ($ngmL^{-1}$)**					
	**<50**	**50-200**	**200-500**	**500-1000**	**1000-10000**	**>10000**
**<50**	**15**	**4**	**1**	**0**	**0**	**0**
**50-200**	**2**	**9**	**1**	**0**	**0**	**0**
**200-500**	**0**	**5**	**5**	**2**	**0**	**0**
**500-1000**	**0**	**0**	**5**	**3**	**2**	**0**
**1000-10000**	**0**	**0**	**1**	**11**	**18**	**2**
**>10000**	**0**	**0**	**0**	**0**	**4**	**22**

Supplementary Table S6. PI-IDA-EPI experimental parameters and Fragmentation of the analytes detected in precursor ion scan

**PI**				**IDA**	**EPI**		
m/z	Range	DP	CE		*m/z*	DP	CE
135			35				
70	100-400	60	25	1. Select 1 to 4 most intense peaks,<br>2. Intensity exceed 1.0E+0.4,<br>3. Always exclude former target ions	80-300	60	25
125			25				

Dibutylone (*m/z* 86, 105, 119, 133, 149, 161, 191, 219, 236)

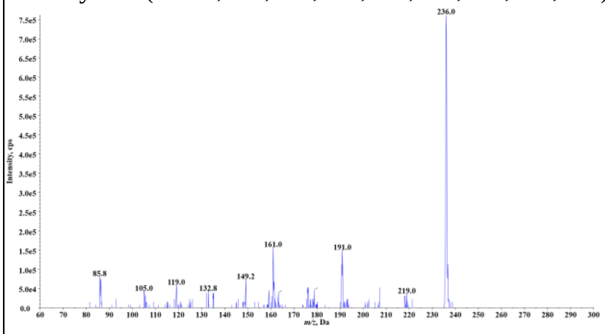

MDPV (*m/z* 126, 135, 149, 175, 205, 276)

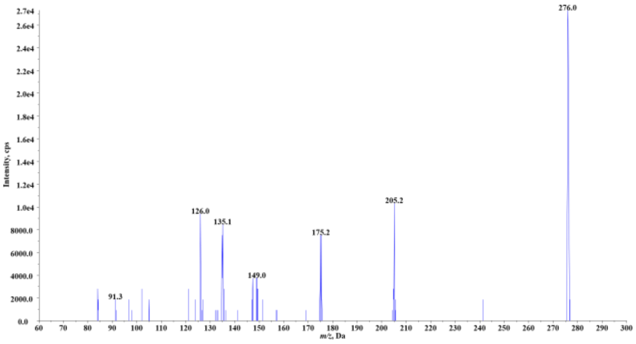

α-PVP (*m/z* 91, 137, 161, 215, 232)

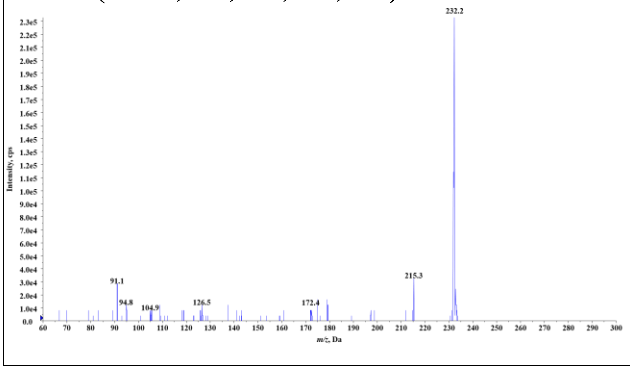

4-chloro-α-PVP (*m/z* 84, 125, 139, 153, 194, 205, 266)

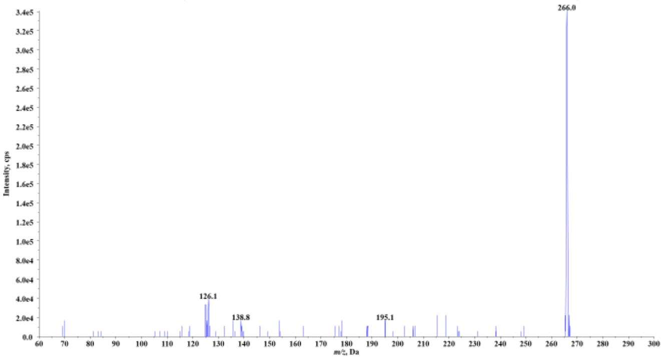

Supplement: Supplementary file 1 — js4c00124_si_001.pdf [file js4c00124_si_001.pdf]
